# Transcriptomics Analysis of* Candida albicans* Treated with Huanglian Jiedu Decoction Using RNA-seq

**DOI:** 10.1155/2016/3198249

**Published:** 2016-04-06

**Authors:** Qianqian Yang, Lei Gao, Maocan Tao, Zhe Chen, Xiaohong Yang, Yi Cao

**Affiliations:** ^1^Zhejiang Hospital of Traditional Chinese Medicine, Zhejiang Chinese Medical University, 54 Youdian Road, Hangzhou 310006, China; ^2^College of Agronomy and Plant Protection, Qingdao Agricultural University, Qingdao 266109, China; ^3^Shandong Provincial Research Center for Bioinformatic Engineering and Technique, School of Life Sciences, Shandong University of Technology, 266 West Cunxi Road, Zibo 255049, China

## Abstract

*Candida albicans* is the major invasive fungal pathogen of humans, causing diseases ranging from superficial mucosal infections to disseminated, systemic infections that are often life-threatening. Resistance of* C. albicans* to antifungal agents and limited antifungal agents has potentially serious implications for management of infections. As a famous multiherb prescription in China, Huanglian Jiedu Decoction (HLJJD,* Orengedokuto* in Japan) is efficient against* Trichophyton mentagrophytes* and* C. albicans*. But the antifungal mechanism of HLJDD remains unclear. In this study, by using RNA-seq technique, we performed a transcriptomics analysis of gene expression changes for* C. albicans* under the treatment of HLJDD. A total of 6057 predicted protein-encoding genes were identified. By gene expression analysis, we obtained a total of 735 differentially expressed genes (DEGs), including 700 upregulated genes and 35 downregulated genes. Genes encoding multidrug transporters such as ABC transporter and MFS transporter were identified to be significantly upregulated. Meanwhile, by pathway enrichment analysis, we identified 26 significant pathways, in which pathways of DNA replication and transporter activity were mainly involved. These results might provide insights for the inhibition mechanism of HLJDD against* C. albicans*.

## 1. Introduction

*Candida albicans* is the most prevalent opportunistic fungal pathogen implicated in superficial mucosal infections as well as invasive disseminated infections, especially in immunocompromised patients [1, 2].* C. albicans* infections are usually treated with antifungal agents, such as azoles, echinocandins, and polyene drugs. Limited by the number of available antifungal targets, the antifungal agents still remain restricted. The azoles are the most widely used drugs for treating pathogenic fungal infections. Sterol 14*α*-demethylase (ERG11) is an ancestral activity of the cytochrome P450 superfamily, which is required for ergosterol biosynthesis in fungi and cholesterol biosynthesis in mammals [3]. As a key enzyme of sterol biosynthesis, Erg11 is the main target for therapeutic azole antifungal drugs [4, 5].

Widespread overuse of azole drugs for decades has led to the occurrence of drug-resistant isolates [6–8]. The prolonged and repeated treatment of OPC (oropharyngeal candidiasis) in AIDS patients has resulted in an increasing frequency of therapy failures caused by the emergence of fluconazole-resistant* C. albicans* strains. In one study, the levels of fluconazole resistance of a series of 17 clinical isolates taken from a single HIV-infected patient who was treated with azoles over 2 years increased over 200-fold [9]. In recent years, the incidence of azole-resistant strains of* C. albicans* has increased, especially the rapid emergence of fluconazole-resistant strains. In the vast majority of countries, far less than 10% of* C. albicans* strains isolated from 1997 to 2001 are resistant to fluconazole [10]. But recent study in China showed that the rate of fluconazole resistance in* C. albicans* was almost 14.1% [11]. In USA, compared with 2008, the proportion of cases identified from 2008 to 2013 from Georgia and Maryland with fluconazole resistance decreased (GA: 8.0% to 7.1%, −10%; MD: 6.6% to 4.9%, −25%), but the proportion of cases with an isolate resistant to an echinocandin increased (GA: 1.2% to 2.9%, +147%; MD: 2.0% to 3.5%, +77%) [12]. So far, several resistance mechanisms of* C. albicans* have been well characterized: alterations in the sterol biosynthesis pathway, mutations in the* ERG11* gene encoding the drug target enzyme, overexpression of the* ERG11* gene, and overexpression of genes encoding efflux pumps [13]. Resistance of* C. albicans* to antifungal agents and limited antifungal agents has potentially serious implications for management of infections.

As a famous multiherb prescription in China, Huanglian Jiedu Decoction (HLJJD,* Orengedokuto* in Japan) is an aqueous extract of 4 herbal materials,* Coptidis Rhizoma*,* Scutellariae Radix*,* Phellodendri Cortex*, and* Gardeniae Fructus *with the ratio of 3 : 2 : 2 : 3. HLJJD was first mentioned in the book* Wai-Tai-Mi-Yao* compiled by Wang Tao in the Tang dynasty (about 752 AD), and it has been widely used in the clinical practice in China and officially listed in the Chinese Pharmacopoeia [14]. HLJJD has been widely used in the treatment of gastrointestinal disorders, inflammation and cardiovascular diseases, and Alzheimer's disease in China [15–17]. Modern pharmacological research also demonstrated multiple biological activities of HLJDD: decreasing levels of plasma glucose and blood lipid in type 2 diabetes mellitus [18, 19]; increasing the cerebral blood flow, inhibiting the platelet aggregation; reducing hypertension and altering the gene expression profiles of spontaneous hypertensive rats [20–23]; reducing hepatic triglyceride accumulation, restraining the preadipocyte differentiation and lipid accumulation, inhibiting the lipid peroxidation, and preventing atherosclerosis [15, 24–26]. Anti-inflammatory effects of HLJDD were also investigated in some papers [18, 27–29]. Moreover, in* Mugil cephalus*, 1% modified HLJDD feeding for 28 days may be an optimal dose to prevent* Lactococcus garvieae* infection and could be used in aquaculture industries. In in vitro study, the modified HLJDD also activated the plasma bactericidal activities [30].

Each herb of HLJDD contains many chemical components. Some papers had reported the content determinations of components contained in HLJDD [31, 32]. HLJDD contains multiple bioactive secondary metabolites, mainly including alkaloids from* Coptidis Rhizoma* and* Phellodendri Cortex*, flavonoids from* Scutellariae Radix*, and terpenes from* Gardeniae Fructus*. There are 4 typical compounds from HLJDD: geniposide, baicalin, berberine, and baicalein [27]. Further study showed that the combination of fluconazole and baicalein or berberine produced potently synergistic action in vitro, while baicalein and berberine showed weak antifungal activity when they were tested alone [33, 34]. Our preliminary work showed that HLJDD is efficient against* Trichophyton mentagrophytes *and* C. albicans *[35]. HLJDD showed its impressive antifungal effect by multitarget and multichannel actions, due to the multiple components. For that reason, the use of HLJDD may be more beneficial to human health in fungal infection treatment as diverse mechanisms showed complementary effects between herbs. But the antifungal mechanism of HLJDD remains unclear.

RNA-seq (deep-sequencing of cDNA) has been used successfully to identify and quantify gene expression at a genome scale level under different conditions or in different cell types. Moreover, it is significantly more sensitive than microarray hybridization approaches [36]. This approach has already been used in* C. albicans *to generate a high-resolution map of the* C. albicans* transcriptome under several different environmental conditions [37]. The effect of berberine chloride on* Microsporum canis* infection was analyzed by the construction of a transcriptome of the* M. canis* cellular responses upon berberine treatment [38]. Therefore, in this study, by using RNA-seq technique, we performed a large-scale analysis of gene expression changes when* C. albicans* was exposed to HLJDD, to better understand how HLJDD inhibits the growth of* C. albicans*.

## 2. Materials and Methods

### 2.1. Strain and Culture Conditions

The* C. albicans* strain used in this study is SC5314 [39].* C. albicans* strains were routinely grown on YPD (1% yeast extract, 2% peptone, and 2% glucose) medium.

### 2.2. Preparation of the Extract of HLJDD

The herbal medicines of modified HLJDD were dried at 40°C for 24 h and then pulverized to powder using a mechanical blender. 0.5%, 1%, and 2% w/w of the powder was prepared and boiled for 30 min with 200 mL of deionized water, and the aqueous extracts were filtered through Whatman number 1 filter paper. The HLJDD residues were also boiled with another 200 mL of deionized water.

### 2.3. Determination of Sensitivity of the SC5314 Strain to HLJDD

Antifungal susceptibility testing was performed by using the CLSI M27-A3 microbroth dilution method [40]. MICs were determined after growth at 30°C for 24 h for HLJDD. MICs were read as the lowest drug concentration producing a prominent decrease in turbidity translating to 100% growth reduction compared with the drug-free control.

### 2.4. Total RNA Extraction

To identify genes in the early response of* C. albicans* to HLJDD, we treated the isolate with HLJDD at 20 mg/mL, the lowest drug concentration producing a prominent decrease in turbidity translating to 100% growth reduction compared with the drug-free control determined above. To extract total RNA, the cells of SC5314 were inoculated into YPD medium and cultured at 30°C overnight. Before SC5314 were harvested for RNA extraction, the culture was treated with HLJDD at 20 mg/mL for 3 h. The untreated culture was used as the control. Total RNA was isolated according to the protocol described by Alison et al. [41].

### 2.5. RNA Sequencing and Assembling

Three independent experiments were performed for the study of either control* C. albicans* or* C. albicans* with HLJDD treatment. Shear cDNA into 300–500 bp fragments using ultrasonic apparatus (Fisher) and purify it with Ampure beads (Agencourt, America). Library of all the samples was constructed according to the procedure of NEBNext® UltraTM RNA Library Prep Kit for Illumina (NEB, America). Sequencing library was checked with Onedrop quantitation, 2% agarose gel electrophoresis detection, and high sensitivity of DNA chip detection. Paired-end sequencing of cDNA was carried out with Illumina Hiseq TM2000. Raw data was filtered by removing reads with adaptor sequences, as well as low quality reads. Then, clean reads were obtained and mapped to reference sequences using SOAP (2.21) [42].

### 2.6. Gene Prediction and Annotation

Trinity software was used to assemble the clean reads into contigs and BLAST (2.2.23) was used to do gene prediction. Predicted sequences (*e*-value < 1.0*e* − 05) were annotated with information from GenBank NR, GO, and KEGG using BLAST2GO (2.2.5). GO classification was conducted using WEGO [43].

### 2.7. Analysis of Differential Expressed Genes

The expression level for each gene is determined by the numbers of reads uniquely mapped to the specific gene and the total number of uniquely mapped reads in the sample. The gene expression level is calculated by using RPKM (Reads Per kb per Million reads) method [36]. Then, NOI seq method was applied to screen differentially expressed genes between two groups, with the threshold of significance as fold change of RPKM ≥ 3 and Probability ≥ 0.8 [44].

### 2.8. Enrichment Analysis of GO and KEGG Pathways

Enrichment analysis was performed by hypergeometric test to find significantly enriched GO terms and KEGG pathways in DEGs. False discovery rate (FDR) of pathways was calculated. The threshold of significance of pathways was set as FDR < 0.05.

### 2.9. Real-Time Quantitative Reverse Transcription- (qRT-) PCR

To evaluate the validation of RNA-seq results, we conducted quantitative real-time (RT) PCR assays for determination of expression of 8 genes. Gene expression levels were calculated using the 2^−ΔΔCt^ method [45]. For each sample, PCR amplifications with primer pair actin-F and actin-R for the quantification of expression of actin gene were performed as a reference. The experiment was repeated 3 times.

## 3. Results

### 3.1. RNA Sequencing and Gene Prediction

Approximately 12,000,000 raw reads were obtained from each sample. After filtering by quality, about 96% clean reads were mapped. Summary of mapping result was shown in Table 1. Using the longest sequence of a subgroup as the unigene as the reference sequence, we got 6057 predicted protein-encoding genes totally. The data have been submitted to NCBI under BioProject accession number PRJNA314910.

### 3.2. Identification and Verification of Differentially Expressed Genes

By using the threshold of significance as fold change of RPKM ≥ 3 and Probability ≥ 0.8, we obtained a total of 735 differentially expressed genes (DEGs), including 700 upregulated genes and 35 downregulated genes (Supporting Information Table S1 in Supplementary Material available online at http://dx.doi.org/10.1155/2016/3198249). The 20 most upregulated genes in response to HLJDD are listed in Table 2.

A total of 8 genes including 7 upregulated and 1 downregulated gene from DGE libraries were selected for real-time PCR analysis to validate the DGE data. The results showed that 8 genes were demonstrated to have a consistent change for both DGE and real-time PCR while actin genes had no significant difference in real-time PCR (Supporting Information Table S2).

### 3.3. Effects of HLJDD Treatment on the Genes Involved in Sterol Biosynthesis

As the most widely used antifungal drugs, azoles can block fungal sterol biosynthesis pathway. Thus, effects of HLJDD on the genes involved in sterol biosynthesis were analyzed in detail. Expression of 23 genes involved in sterol biosynthesis was detected in the RNA-seq analysis, and expression of 8 genes showed a more than 2-fold increase after* C. albicans* was treated with HLJDD; only the genes encoding sterol 24-C-methyltransferase (*ERG6*) and C-8 sterol isomerase (*ERG2*) were upregulated by more than 3 times (Table 3). None of these 23 genes was downregulated significantly (Probability > 0.8) by HLJJD.

### 3.4. Effects of HLJDD Treatment on the Genes Encoding Multidrug Transporters

In* C. albicans*, upregulation of multidrug transporter genes is one of the well-documented mechanisms of resistance to azole antifungal agents [9, 46–48]. Two families of multidrug transporters, the ABC (ATP-binding cassette) transporter family (Cdr1p and Cdr2p) and the major facilitator superfamily (MFS, CaMdr1p), have been shown to be involved in resistance to azole antifungal agents [47, 48]. Thus, we also paid attention to the multidrug transporter genes. In genome sequences of* C. albicans*, a total of 36 genes are annotated as multidrug transporters. In this study, expression of 32 genes was detected by the RNA-seq, and 7 genes were identified to be significantly upregulated more than 3 times by HLJDD treatment (Table 4), including* CDR2* (*Candida* Drug Resistance) from the family of ABC transporters. Cdr2 has been shown as the principal mediators of resistance to azoles due to transport phenomena [47, 48].

### 3.5. Enrichment Analysis of GO and KEGG Pathways

**Figure 1:**
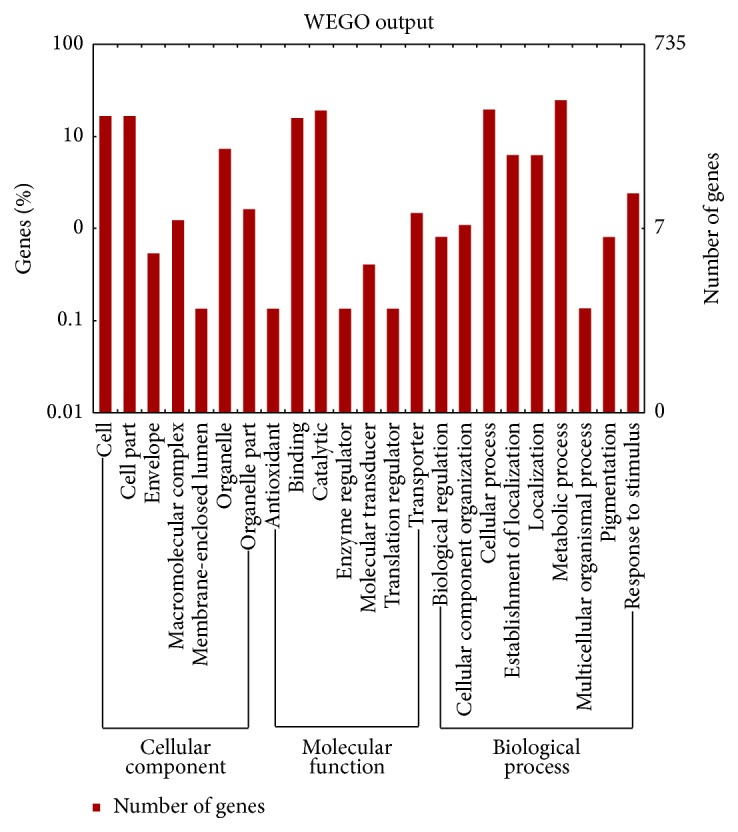
Functional categories of genes in* C. albicans* in response to HLJDD.

GO and KEGG assignments were used to classify the genes in the response of* C. albicans* to HLJDD. By GO classification analysis, the percentage and distribution of top-level GO terms were portrayed in the 3 categories: (A) cellular component; (B) molecular function, and (C) biological process (Figure 1). A high percentage of genes were assigned to “cell,” “cell part,” “binding,” “catalytic,” “cellular process,” and “metabolic process” (Figure 1).

By enrichment analysis, with FDR < 0.05, 23 significant GO terms and 3 significant KEGG pathways were identified (Supporting Information Table S3). These significant pathways were mainly associated with DNA replication and transporter activity. The maps with highest unigene representation were meiosis (cal04113; 23 unigenes), followed by cell cycle (cal04111; 23 unigenes), and DNA replication (cal03030; 11 unigenes).

## 4. Discussion

*C. albicans* is the most prevalent opportunistic fungal pathogen causing superficial to systemic infections in immunocompromised individuals [1, 2]. The concomitant use of drugs and the lack of available drugs frequently result in the occurrence of drug-resistant isolates and strains display multidrug resistance (MDR). In search of novel fungicides, efficiency of medicinal plants against fungi has been reported, but studies on their underlying mechanisms are very few [49]. In this study, we explored a famous multiherb prescription in China, Huanglian Jiedu Decoction (HLJJD,* Orengedokuto* in Japan), for its antifungal potential. Our preliminary work showed that HLJDD is efficient against* Trichophyton mentagrophytes *and* C. albicans* [35]. HLJDD showed its impressive antifungal effect by multitarget and multichannel actions, but studies on the underlying mechanisms are very few. To determine the antifungal mechanism of HLJDD against* C. albicans*, we performed a large-scale analysis of gene expression changes when* C. albicans* was exposed to HLJDD, to better understand how HLJDD inhibits the growth of* C. albicans*. Due to the multiple components of HLJDD, it is most likely that the antifungal effect is multitarget and multichannel actions. KEGG analysis suggested that 3 cellular functions were affected in* C. albicans* upon HLJDD treatment, including meiosis, cell cycle, and DNA replication. Most genes (56 genes) involved in the 3 cellular functions were upregulated excepted for 1 gene, potential hexose transporter (XP_719596.1). Among these genes, Spo22 (also called Zip4) (XP_718811.1) was upregulated obviously upon HLJDD treatment. Zip4/Spo22 was shown to be a central protein of the SICs (synapsis initiation complexes), from which the polymerization of the transverse filament proceeds. In* S. cerevisiae*, Zip4/Spo22 was identified as a member of the ZMM group of proteins that also includes Zip1, Zip2, Zip3, Msh4, Msh5, and Mer3 which together control the formation of class I COs [50–52]. In* Arabidopsis thaliana*, Zip4/Spo22 function in class I CO formation is conserved with budding yeast. However, mutation in AtZIP4 does not prevent synapsis, showing that both aspects of the Zip4 function (i.e., class I CO maturation and synapsis) can be uncoupled [51].

Azoles are the most widely used antifungal drugs, which target on cytochrome P450 lanosterol 14*α*-demethylase encoded by the* ERG11* gene. In* Fusarium graminearum*, using a deep serial analysis of gene expression (DeepSAGE) sequencing approach, the transcriptional response of* F. graminearum* to tebuconazole (a widely used azole fungicide) was profiled. Expression of 23 genes involved in sterol biosynthesis was detected in the DeepSAGE analysis, and expression of 9 genes showed a more than 5-fold increase after the fungus was treated with tebuconazole. None of these 23 genes was downregulated by more than 5 times by tebuconazole [53]. Thus, effects of HLJDD on the genes involved in sterol biosynthesis were analyzed in detail. Expression of 23 genes involved in sterol biosynthesis was detected in the RNA-seq analysis, and expression of 8 genes showed a more than 2-fold increase after the fungus was treated with HLJDD, only the genes encoding sterol 24-C-methyltransferase (*ERG6*) and C-8 sterol isomerase (*ERG2*) were upregulated by more than 3 times (Table 3). None of these 23 genes was downregulated significantly (Probability > 0.8) by HLJJD. These results indicate that HLJDD might also affect sterol biosynthesis of* C. albicans*.

Overexpression of multidrug resistance efflux transporter genes in several fungi was found to be correlated with azole resistance [54]. In* C. albicans*, a number of efflux transporter genes have been cloned and characterized. Two families of multidrug transporters, the ABC (ATP-binding cassette) transporter family (Cdr1p and Cdr2p) and the major facilitator superfamily (MFS, CaMdr1p), have been shown to be involved in resistance to azole antifungal agents [47, 48]. Expression of 32 genes out of 36 genes annotated as multidrug transporters in genome sequences of* C. albicans *was detected by the RNA-seq sequencing. Expression of 13 genes was upregulated by more than 2 times by HLJDD; meanwhile, only 2 genes were significantly downregulated including* CDR4*. In addition, expression of only 4 genes was upregulated by more than 3 times by HLJDD, including* CDR2*, which plays an important role in azole resistance (Table 4). The upregulated expression of these genes may be related to efflux of HLJJD, which provides supporting evidence to previous studies on expression level.

Previous study examined changes in the gene expression profile of* C. albicans* following exposure to representatives of the 4 currently available classes of antifungal agents, the azoles (ketoconazole), polyenes (amphotericin B), echinocandins (caspofungin), and nucleotide analogs (5-flucytosine). And the data showed that none of the differentially regulated genes found exhibited similar changes in expression for all 4 classes of drugs. Thus, the response of* C. albicans* to different drugs seems to be highly specific [55]. Ketoconazole exposure increased the expression of genes involved in lipid, fatty acid, sterol metabolism, and several genes associated with azole resistance, including* CDR1* and* CDR2* [56]. It is surprising that HLJDD increased the expression of genes involved in sterol metabolism and azole resistance (*CDR1* and* CDR2*). Considering the similarity of expression changing pattern, it is possible that HLJDD affects sterol metabolism. And further experiments are required to confirm this hypothesis.

## 5. Conclusions

In conclusion, we performed a transcriptomics analysis of gene expression changes for* C. albicans* under treatment of HLJDD using RNA-seq technique. Overall, a total of 6057 predicted protein-encoding genes were identified. Further gene expression analysis revealed a total of 735 differentially expressed genes (DEGs), including 700 upregulated genes and 35 downregulated genes. Intensive bioinformatics analysis identified 26 significant pathways, and DNA replication and transporter activity were mainly involved. In addition, genes encoding multidrug transporters such as ABC transporter and MFS transporter were identified to be significantly upregulated. Overall, the results from this study might provide insights in understanding of the mechanisms for the response of* C. albicans *to HLJJD. Furthermore, this work demonstrates the potential utility of the RNA-seq technique in antifungal studies.

## Supplementary Material

Table S1: 735 differentially expressed genes (DEGs).
Table S2: A list of primers used in real-time PCR analysis and comparison in the changes of gene expression.
Table S3: Significant pathways of DEGs.

## Figures and Tables

**Table 1:** Summary of reads in *C. albicans* with or without HLJDD treatment.

Sample	Total Reads	Total mapped reads	Mapping percentage
Ca_CK_1	12,377,083	11,840,278	95.66%
Ca_CK_2	11,758,367	11,313,372	96.22%
Ca_CK_3	12,283,182	11,830,964	96.32%
Ca_HT_1	12,406,841	11,924,995	96.12%
Ca_HT_2	11,803,831	11,338,558	96.06%
Ca_HT_3	12,212,721	11,727,140	96.02%

**Table 2:** The 20 most upregulated genes in response to HLJDD treatment.

Standard or systematic name in CGD	ID in GenBank	Annotation	Size	log_2_⁡ ratio	Probability
C7_01060W_A	XP_720301.1	Hypothetical protein	142 aa	11.25	0.81
C5_04240C_A	XP_721977.1	Hypothetical protein	103 aa	11.21	0.80
C1_03880C_A	XP_711956.1	Hypothetical protein	120 aa	9.85	0.95
C7_01130C_A	XP_712469.1	Hypothetical protein	146 aa	9.42	0.92
*PGA39*	EEQ43586.1	Predicted protein	288 aa	9.32	0.85
C1_12040W_A	XP_716393.1	Hypothetical protein	143 aa	9.31	0.92
CR_01870C_A	XP_718251.1	Hypothetical protein	196 aa	8.90	0.85
CR_04980C_A	XP_711981.1	Hypothetical protein	193 aa	8.87	0.92
*LIP10*	XP_723508	Secretory lipase 10	465 aa	8.85	0.81
C3_01010W_A	XP_718606.1	Hypothetical protein	102 aa	8.67	0.90
C4_06150C_A	EEQ44911.1	Tat binding protein 1-interacting	175 aa	8.27	0.99
C7_03900W_A	XP_715240.1	Hypothetical protein	109 aa	7.96	0.94
CR_07970C_A	XP_714226.1	Hypothetical protein	119 aa	7.91	0.81
CR_06990W_A	XP_712676.1	Transcription activator	865 aa	7.84	0.88
C5_02090W_A	EEQ43139.1	Predicted protein	100 aa	7.84	0.84
*SPO22*	XP_718811.1	Meiosis specific protein	566 aa	7.73	0.96
CR_07550C_A	XP_710398.1	Hypothetical protein	101 aa	7.72	0.82
C3_02250C_A	XP_721699.1	Hypothetical protein	162 aa	7.71	0.85
C7_01060W_A	XP_718305.1	Hypothetical protein	111 aa	7.70	0.89

**Table 3:** Response to HLJDD of the genes involved in ergosterol biosynthesis.

Standard or systematic name in CGD	ID in GenBank	Annotation	log_2_⁡ ratio	Probability
*ERG1*	XP_711894.1	Squalene monooxygenase	2.13	0.95
*ERG2*	XP_718886.1	C-8 sterol isomerase	3.23	0.94
*ERG3*	XP_713577.1	C-5 sterol desaturase	1.84	0.94
*ERG4*	XP_717662.1	Sterol C-24 (28) reductase	−0.34	0.65
*ERG5*	XP_716933.1	Cytochrome P450 61	2.02	0.97
*ERG6*	XP_721588.1	Sterol 24-C-methyltransferase	3.33	0.97
*ERG7*	XP_722471.1	2,3-Oxidosqualene-lanosterol cyclase	−0.23	0.38
*ERG8*	XP_722678.1	Phosphomevalonate kinase	−0.01	0.03
*ERG9*	XP_714460.1	Squalene synthetase	−0.60	0.77
*ERG10*	XP_710124.1	Acetyl-CoA acetyltransferase IA	0.96	0.91
*ERG11*	XP_716761.1	Cytochrome P450 51	2.02	0.97
*ERG12*	XP_723305.1	Mevalonate kinase	0.97	0.85
*ERG13*	XP_716446.1	Hydroxymethylglutaryl-CoA synthase	2.30	0.97
*MVD1/ERG19*	XP_718960.1	Diphosphomevalonate decarboxylase	0.04	0.12
*ERG24*	XP_710205.1	Delta(14)-sterol reductase	2.53	0.93
*ERG25*	XP_713420.1	C-4 methylsterol oxidase	1.31	0.91
	XP_722703.1	C-4 methylsterol oxidase	1.02	0.92
*ERG26*	XP_715564.1	C-3 sterol dehydrogenase/C-4 decarboxylase	0.23	0.39
*ERG27*	XP_717865.1	3-Keto sterol reductase	1.67	0.90
*ERG28*	XP_717865.1	Hypothetical protein	0.38	0.68
*HMG1*	XP_713636.1	Hydroxymethylglutaryl-CoA reductase	2.36	0.96
*IDI1*	XP_720295.1	Isopentenyl-diphosphate delta-isomerase	−0.09	0.24
*CYB5*	XP_720295.1	Cytochrome b5	−0.27	0.62

**Table 4:** Response to HLJDD of the genes involved in multidrug resistance of *C. albicans.*

Standard or systematic name in CGD	ID in GenBank	Annotation	log_2_⁡ ratio	Probability
*CDR1*	XP_723062.1	Multidrug resistance protein CDR1	2.42	0.99
*CDR2*	XP_723022.1	Multidrug resistance ABC transporter	**5.32**	0.99
*CDR3*	XP_441615.1	N terminal 2/3 of opaque-specific ABC transporter	0.75	0.67
*CDR4*	XP_717543.1	Potential ABC transporter	−2.49	0.99
*ATM1*	XP_712090.1	Potential mitochondrial ABC transporter similar to* S. cerevisiae* ATM1	0.79	0.76
*HST6*	XP_716101.1	Potential ABC transporter similar to *S. cerevisiae* STE6	**5.44**	0.88
*MDL1*	XP_718280.1	Potential ABC transporter similar to *S. cerevisiae* mitochondrial inner membrane MDL1	0.81	0.79
*MLT1*	XP_717637.1	Vacuolar multidrug resistance ABC transporter	1.75	0.93
*MDR1*	XP_719165.1	Major Facilitator Transporter	0.63	0.77
CR_04620C_A	XP_717510.1	MFS transporter, DHA1 family, multidrug resistance protein	**4.46**	0.91
*SGE11*	*XP_715705.1*	*Potential MFS-MDR transporter*	*1.31*	*0.84*
C1_10710C_A	XP_714012.1	MFS transporter, DHA2 family, multidrug resistance protein	**5.1**	0.90
C3_03070W_A	XP_720131.1	MFS transporter, DHA2 family, multidrug resistance protein	0.47	0.66
*NAG4*	XP_712435.1	MFS transporter, DHA1 family, multidrug resistance protein	**5.83**	0.77
*TPO41*	XP_717426.1	MFS transporter, DHA1 family, multidrug resistance protein	2.34	0.95
C6_01870C_A	XP_716705.1	MFS transporter, DHA1 family, multidrug resistance protein	2.59	0.94
*NAG3*	XP_712434.1	MFS transporter, DHA1 family, multidrug resistance protein	2.8	0.85
C1_10200C_A	XP_723572.1	MFS transporter, DHA1 family, multidrug resistance protein	1.23	0.91
C2_02570W_A	EEQ45693.1	MFS transporter, DHA1 family, multidrug resistance protein	1.34	0.78
*TPO3*	XP_723233.1	MFS transporter, DHA1 family, multidrug resistance protein	−0.95	0.85
*HOL1*	XP_721489.1	MFS transporter, DHA1 family, multidrug resistance protein	2.0	0.85
CR_01340W_A	XP_718285.1	MFS transporter, DHA1 family, multidrug resistance protein	**3.93**	0.93
*HOL4*	XP_712971.1	MFS transporter, DHA1 family, multidrug resistance protein	0.88	0.81
C3_03440C_A	XP_720169.1	Potential drug or polyamine transporter	3.44	0.95
*TPO2*	XP_715197.1	Potential drug or polyamine transporter	2.31	0.82
*QDR3*	XP_714342.1	Potential multidrug resistance transporter	2.33	0.87
C2_00540W_A	XP_719644.1	Potential MATE family drug/sodium antiporter	−0.28	0.56
C7_03590C_A	EEQ47129.1	Multidrug resistance protein, MATE family	0.16	0.26
C1_00830W_A	XP_718985.1	Potential MATE family drug/sodium antiporter	0.44	0.34
CR_10640W_A	XP_719407.1	Multidrug resistance protein, MATE family	**3.15**	0.96
*QDR2*	XP_714698.1	Potential quinidine/multidrug transporter	1.63	0.94
*FLU1*	XP_721413.1	Multidrug efflux transporter	1.76	0.91
